# Discovery of therapeutic targets in cardiovascular diseases using high-throughput chromosome conformation capture (Hi-C)

**DOI:** 10.3389/fgene.2025.1515010

**Published:** 2025-03-13

**Authors:** Quan Zheng, Ying Liu, Minghao Guo, Xin Zhang, Qingbin Zhang, Xi-Yong Yu, Zhongxiao Lin

**Affiliations:** ^1^ School of Pharmacy, Macau University of Science and Technology, Taipa, China; ^2^ Department of Pharmacology, School of Pharmacy, Guangzhou Xinhua University, Guangzhou, China; ^3^ Guangzhou Municipal and Guangdong Provincial Key Laboratory of Molecular Target & Clinical Pharmacology, The NMPA and State Key Laboratory of Respiratory Disease, School of Pharmaceutical Sciences, Guangzhou Medical University, Guangzhou, China

**Keywords:** cardiovascular system, epigenetic, heart disease, Hi-C, therapeutic targets, three-dimensional structure

## Abstract

Epigenetic changes have been associated with several cardiovascular diseases. In recent years, epigenetic inheritance based on spatial changes has gradually attracted attention. Alterations in three-dimensional chromatin structures have been shown to regulate gene expression and influence disease onset and progression. High-throughput Chromosome Conformation Capture (Hi-C) is a powerful method to detect spatial chromatin conformation changes. Since its development, Hi-C technology has been widely adopted for discovering novel therapeutic targets in cardiovascular research. In this review, we summarize key targets identified by Hi-C in cardiovascular diseases and discuss their potential implications for epigenetic therapy.

## Introduction

Cardiovascular diseases (CVDs) are one of the deadliest diseases in the world and seriously threaten people’s health, with a particularly alarming impact nations with moderate economic status. The mortality rate of CVDs is even higher than that of cancer. However, in high-income countries, improvements in the prevention and treatment of CVDs have led to a lower mortality rate for CVDs than for cancer (Timmis et al., 2022). The Institute for Health Metrics and Evaluation (IHME) classifies cardiovascular disease, as including mortality and morbidity, into 11 distinct cardiovascular conditions. These primarily include ischemic heart disease, cerebrovascular accidents, hypertensive cardiac disorders, and congestive heart failure (TIfHMa, 2021). Approximately 330 million individuals in China are believed to be afflicted with cardiovascular diseases, with the nation grappling with the compounding effects of an aging demographic and a consistent increase in the prevalence of metabolic risk factors (Sheng-Shou, 2022). Researchers are still finding new targets for the prevention and treatment of CVDs. Proprotein convertase subtilisin/kexin type 9 (PCSK9) inhibitors block the binding of the PCSK9 protein to LDL receptors on the cell surface and prevent their degradation. This action allows more LDL receptors to be recycled back to the cell surface. As a result, there is increased uptake of LDL cholesterol, which reduces its levels in the bloodstream. Because they are effective at lowering LDL cholesterol over the long term, PCSK9 inhibitors offer a good alternative to statin therapy (McGettigan and Ferner, 2017; Burnett and Hooper, 2018). Calcium channel antagonists (CCAs) relieve arterial constriction by blocking calcium influx and binding to specific sites on voltage-sensitive calcium channels, making them the preferred treatment for hypertension (Godfraind, 2014; Carey et al., 2022). These examples show that treatment targets are very important for cardiovascular treatment. Therefore, the discovery of new targets is highly important for the study of cardiovascular diseases. In this study, we focused on the discovery of Hi-C-based therapeutic targets. Paul et al. captured Hi-C data from B cells and T cells to identify high-confidence candidate causal genes that could be used to treat these diseases (Martin et al., 2019).

Modern genetics shows that the use of genetic information to guide personalized medicine has great potential. There are many sequencing technologies, such as ChIP-seq (chromatin immunoprecipitation sequencing), ATAC-seq (assay for transposase-accessible chromatin using sequencing), methyl-seq (DNA methylation sequencing), and RNA-seq (transcriptome sequencing) (Goodwin et al., 2016). However, when detecting epigenetic modifications, the focus is on identifying changes in gene expression caused by changes in the spatial conformation of chromatin, such as long-range interactions. Few existing techniques are able to perform detection at high resolution. The interaction of chromosome spatial structure can regulate gene expression, leading to changes in the epigenetic state (Dekker, 2008). The 3D structure of chromatin is highly complex. Recently, an increasing number of studies have shown that gene regulation, including DNA replication, transcription, and translation, can be affected by the 3D structure of chromatin to some extent. Therefore, understanding the 3D structural epigenetics of chromatin in cardiovascular disease models is important, and clarifying the 3D structure of chromatin can improve disease detection.

Currently, 3D genomic chromatin conformation capture technologies and their derivatives, known as C technologies, can be broadly divided into 3C (one-to-one), 4C (one-to-many), 5C (many-to-many), and Hi-C (all-to-all) methods. Hi-C technology is a 3C technology. Compared with traditional 3C approaches, the Hi-C method has improved the resolution and accuracy, offering a more comprehensive view of interactions among different regulatory sequences. Hi-C is good at detecting extensive chromatin contacts in an unbiased, genome-wide manner (van Berkum et al., 2010) and can be used to study the spatial relationships of chromatin DNA, thereby obtaining high-resolution chromatin regulatory element interaction maps. Hi-C and other detection technologies open the door to explore the 3D world of chromatin, and these technologies are highly important for this research. Research indicates that the three-dimensional architecture of chromatin constitutes an epigenetic pathway influencing gene expression patterns in the development of tumors and the emergence of cancer stem cells (Feng and Pauklin, 2020). Hi-C technology has been used to detect the 3D chromatin topology of humans and mitochondria and its impact on mitochondrial diseases (Feng et al., 2021a). Hi-C technology can be used to guide the research and detection of diseases, such as cancer (Feng et al., 2021b). In other words, Hi-C technology can be used to analyze genomic changes in the occurrence of cardiovascular diseases from the perspective of 3D spatial structure, not only from the perspective of linear structure, to clarify the pathogenesis of cardiovascular diseases and predict their occurrence.

**FIGURE 1:**
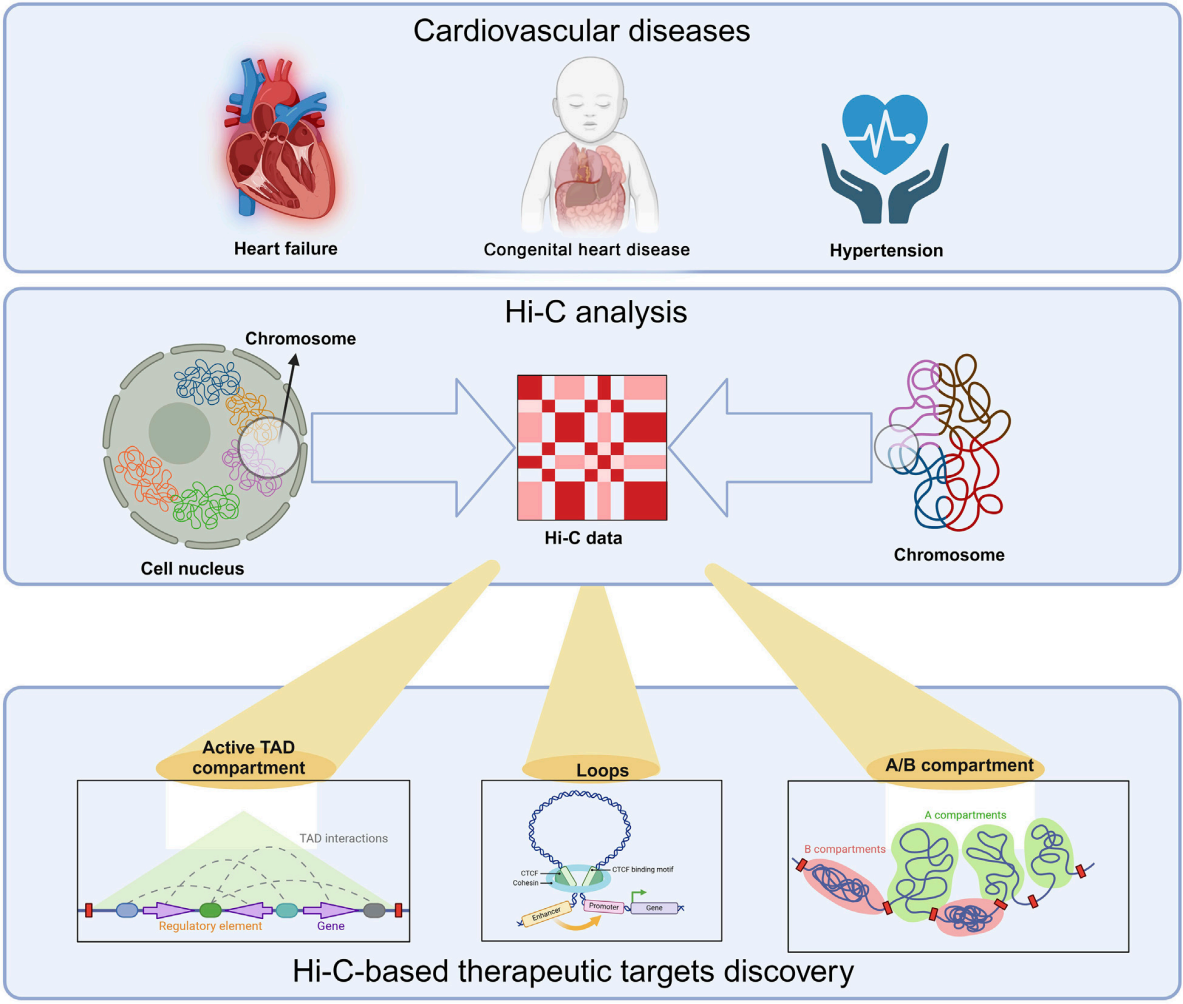
Hi-C-based therapeutic targets discovery. Hi-C and related technology have been used in cardiovascular diseases, such as, heart failure, congenital heart disease and hypertension. Three key contents are crucial in this technology: A/B compartments, topologically associating domains (TADs), and chromatin loops. A/B compartments (euchromatin/heterochromatin) exhibit tissue, temporal, and state specificity. They can transition between different tissues, developmental stages, or disease states. This transition is related to gene expression regulation. When a chromosomal region undergoes an A/B compartment conversion, it affects the transcriptional activity of the associated genes. Generally, genes in regions converting from B compartment to A compartment tend to be upregulated, while those shifting from A compartment to B compartment are downregulated. TADs are segments of DNA with folded structures, serving as the basic organizational units of the genome in three-dimensional space. The interaction frequency within a TAD is significantly higher than that between adjacent regions. TAD structures exhibit a degree of conservation across various contexts, including tissues, developmental stages, and states, while also displaying dynamic changes. Loops are formed through long-range interactions in DNA mediated by proteins such as CTCF. These loop structures also undergo dynamic changes across different contexts. The anchor points of loops often include promoters, enhancers, and silencers. Dynamic changes in loop structures, such as formation or disappearance, can significantly influence gene regulation.

In recent years, many studies have investigated how 3D chromatin structure affects the pathogenesis of cardiovascular diseases. This review aims to summarize the integration of Hi-C data and that cardiovascular diseases (heart failure, congenital heart disease, and hypertension) are highly likely to cause cardiovascular diseases. (Figure 1), explain the importance of Hi-C technology for discovering new targets, and discuss whether Hi-C has deeper applications in exploring the pathogenesis of cardiovascular diseases and detecting diseases.

## Origin and usage of Hi-C technology

**FIGURE 2:**
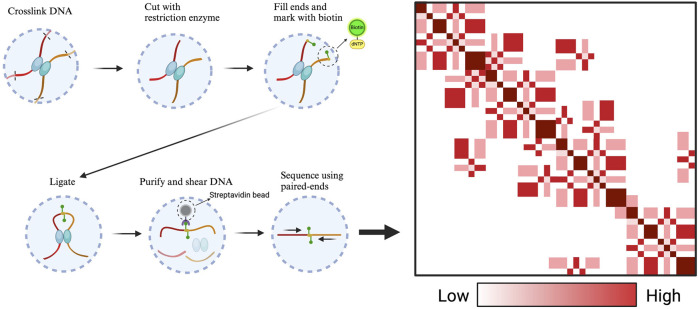
The processing flow of Hi-C technology. Crossklinked DNA is cut with restriction enzyme and then use the sequencing technology to detect the 3D space structure. The intensity of interactions between chromatin DNA fragments is represented in the form of a heatmap. Both the horizontal and vertical axes represent chromatin position intervals, forming a two-dimensional interaction matrix. The different colors represent the intensity or salience of the interaction area.

**FIGURE 3:**
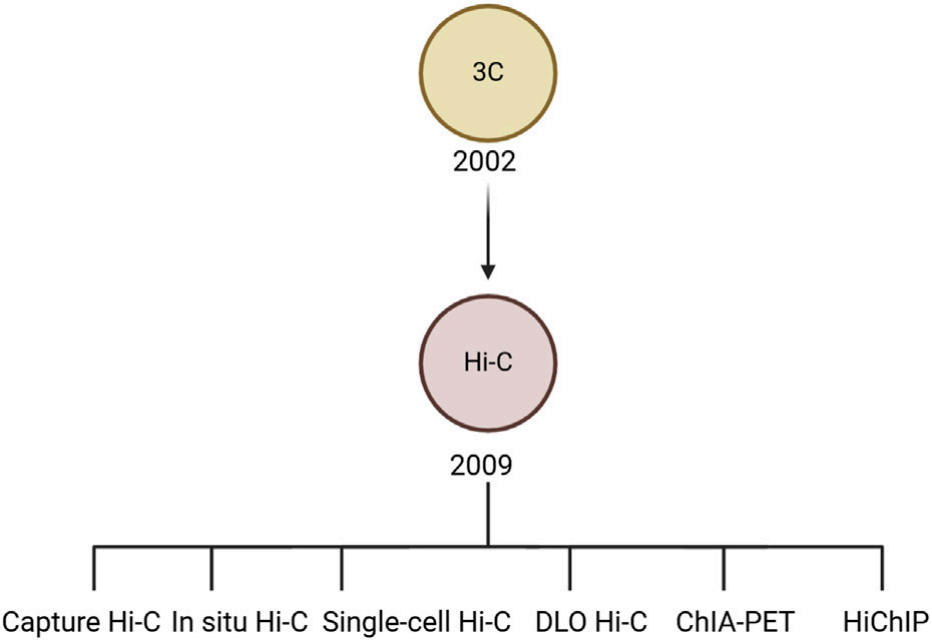
The history of Hi-C technology. 3C was developed in 2002 and Hi-C was created in 2009. Based on Hi-C, several methods were also developed, such as capture Hi-C, single-cell Hi-C, *in situ* Hi-C, DLO Hi-C, ChIA-PET, and HiChIP.

The three-dimensional architecture of chromatin plays a crucial role in gene expression. Its structure is made up of nucleosomes, where DNA is damaged and connected by DNA bridges. DNA in these coiled regions is less actively transcribed than the DNA segments that serve as bridges. This intricate network creates a complex topology that influences gene activity. To address the challenges posed by this complex topology and its impact on gene expression, the 3C technique was introduced. Initially, developed by Job Dekker in 2002, 3C was used to study interactions between nearby genomic regions across bacterial and human chromosomes, including those on the same or different chromosomes (Dekker et al., 2002). The principle of 3C technology is that it uses cross-linking of proteins in the nucleus to analyze whole-genome interactions, allowing inference about the physical interactions of chromosomes, their nature, and their three-dimensional conformation. However, 3C is limited in the size of interacting fragments and cannot be used to screen fragments directly across the whole genome, so it is necessary to predict interacting fragments in advance. Consequently, the 3C methodology is characterized by its limited output, time-intensive nature, and extensive processing requirements. Building on 3C, Hi-C technology offers a high-throughput and comprehensive approach for mapping the spatial organization of genomic DNA across the entire genome, enabling the unbiased differentiation of interactions between genes and their regulatory elements (van Berkum et al., 2010). Compared with 3C, capture Hi-C technology has improved accuracy and precision and can completely show the interaction between each regulatory element (Figure 2). However, Hi-C capture requires a large number of cells, which are not easy to obtain for relatively precious cells. Therefore, single-cell capture Hi-C technology was developed. However, extracting single cells from human tissues, especially living bodies, is difficult. Thus, Dip-C technology was developed to improve the throughput of parallel processing of single cells. After that, ChIA-Drop technology (a strategy for multi-chromatin interaction analysis) was also developed to address the shortcomings of Hi-C technology in cell library construction (Zheng et al., 2019). Currently, common Hi-C-derived techniques include capture Hi-C, single-cell Hi-C, *in situ* Hi-C, DLO Hi-C, ChIA-PET, and HiChIP, etc. (Figure 3).

Moreover, Hi-C detects not only the three-dimensional structure but also the methylation, carboxylation, and phosphorylation of DNA “bridges” and coiled segments. This is because these chemical changes can alter the three-dimensional structure of chromatin and cause differences in gene expression. If we understand the changes in the three-dimensional structure of a chromosome (and its related genes) that can cause diseases, we can find ways to overcome these diseases. Studies have shown that parts of genes linked to heart disease often interact with distant DNA regions. These regions have many specific protein binding sites and special marks on the DNA package. These interactions help confirm a detailed 3D map of the genome (Hi-C profile), showing how genes are organized and how their activity changes (Montefiori et al., 2018). In other words, Hi-C technology can be used to determine the promoters of CVD-related genes and accurately draw promoter maps. Although the promoter is only a small fragment of DNA, it is possible to infer the changed genes. In combination with experimental verification, several potential targets can be identified.

## The target discovery ability of Hi-C technology in cardiovascular diseases

### Heart failure

In the realm of heart health, the PCHi-C approach has identified over 500 genomic regions linked to cardiovascular disease susceptibility. These regions are mostly found in non-coding segments that are distant from the actual genes (Wang and Bennett, 2012). Changes in the epigenome structure impact the phenotype of cardiomyocytes. Using mice with a targeted deletion of CTCF—a protein essential for chromatin architecture—researchers have gained new insights into intrinsic chromatin organization, which has significant implications for the field of epigenetic treatments. These findings indicate that comprehensive restructuring of the chromatin architecture is a primary driver of heart failure (Rosa-Garrido et al., 2017). In this study, chromatin capture, DNA sequencing, and RNA-seq were used to determine the endogenous structure of the cardiomyocyte epigenome. Moreover, Hi-C technology and DNA sequencing analysis revealed interactions within the cardiomyocyte genome at a 5 kb resolution. These cardiomyocytes may share the same regulatory mechanisms as some normal muscle cells (which may also be heterogeneous), and understanding these mechanisms is important for the prevention and treatment of this disease in the future.

Hi-C technology can be applied to genome-wide association and multiomics (such as genome, proteome, transcriptome, epigenome, metabolome and microbiome) analyses. Hi-C was used to scrutinize the 5-kilobase segment surrounding the cardiomyocyte target gene and its neighboring gene-promoter interactions, and the findings revealed that the ATAC-seq peak corresponded to the promoter region of the ACTN2 gene, a gene now recognized as being implicated in heart failure (Sabino et al., 2022; Arvanitis et al., 2020). By identifying the promoter and gene regulation in the 3D structure, we can link heart failure with genomics, thereby expanding our understanding of the potential biological impact of heart failure pathogenic genes. These cardiomyocytes may share the same regulatory mechanisms as some normal muscle cells (itwhich may also be heterogeneous), and explaining these mechanisms is important for the prevention and treatment of this disease in the future.

The LMNA E161K mutation in cardiomyopathy causes abnormal gene expression and improper positioning of chromosome 13. In fibroblasts with LMNA mutations, the entire region of chromosome 13 moves toward the center of the nucleus (Mewborn et al., 2010). In one study, researchers looked at cases of dilated cardiomyopathy caused by mutations in lamin A. They used Hi-C technology to examine heart muscle cells (hiPSC-CMs) derived from human induced pluripotent stem cells. They a lack of haploid mutations in the lamin A/C gene. This finding suggests that, contrary to some hypotheses, global errors in chromosomal compartmentation are not the primary pathogenic mechanism in heart failure due to lamin A/C haploinsufficiency, as concluded by Bertero et al. (2019) (Bertero et al., 2019). Laminin is a structural component of the nuclear lamina (NL) that regulates genome organization and gene expression. By integrating Hi-C with fluorescent *in situ* hybridization (FISH) and examining lamina-associated domains (LADs), scientists have reported that the absence of laminin in mouse embryonic stem cells (ESCs) results in the expansion or partitioning of particular LADs. An in-depth study on laminin deficiency revealed a significant link between shifts in gene transcription and alterations in the interactions of active and inactive chromatin domains (Zheng et al., 2018). Gaining insights into the mechanisms of laminin offers new perspectives for identifying potential treatment approaches for heart failure, including conditions such as dilated cardiomyopathy and cardiac conduction abnormalities such as those associated with missense mutations of genes (Fatkin et al., 1999). However, further experiments are needed to identify related genes, just as we found that DNA methylation disorders are present in heart failure, and some experimental studies still need to be performed.

### Congenital heart disease

With the development of epigenetics and 3D genomics, the molecular mechanisms of heart disease are becoming more closely linked to the 3D structure of chromatin and its regulation. Congenital heart disease (CHD) is a heart malformation caused by problems in the development of the heart and large blood vessels during the fetal stage. Despite CHD being the most common type of survivable birth defect in humans, the exact causes of most CHDs are still not well understood. Epigenetic modifications and changes in chromatin structure play key roles in heart development and disease (Williams et al., 2019; Yuan et al., 2013). Hi-C technology, which is a high-throughput method for studying the 3D structure of chromatin, offers a new way to understand the molecular mechanisms of CHD.

After decades of genome-wide association studies (GWASs), the vast majority of genetic variation associated with disease has been noncoding and is often located in intergenic regions. However, GWASs typically identify only genetic variations associated with diseases and cannot directly determine how these variations affect gene expression and disease occurrence, making them difficult to interpret and thus not easily interpret. Alterations in the epigenetic landscape within non-dividing cells are not typically passed down through generations; however, they can influence gene expression and functionality at the cellular level (Ling and Rönn, 2019). Since most of these genetic variants are believed to modulate gene expression in nearby areas, combining GWAS with 3C-derived data could reveal direct interactions between disease-associated variants and their corresponding target sites (Risca and Greenleaf, 2015). Hi-C technology can be used to study the pathogenic genes of CHD by detecting the role of regulatory genes such as promoters and enhancers in the three-dimensional structure.

In CHD, the expression of key genes such as NKX2.5 and GATA4 is regulated by promoters and enhancers. For example, NKX2.5 can upregulate GDF1 expression by binding to the GDF1 promoter (Gao et al., 2019). Additionally, two human TBX5 promoter regions (upstream of exons 1 and 2) likely regulate the transcription of different transcript variants (Varela et al., 2021). Mutations in these genes or abnormalities in their regulatory elements can lead to abnormal heart development. Moreover, DNA methylation can affect the activity of gene promoters, thereby influencing gene expression. Studies have identified large genomic regions that are differentially methylated during cardiomyocyte development and maturation, indicating that dynamic DNA methylation regulates cardiomyocyte development, maturation, and disease. CHD patients have a specific DNA methylation profile. Abnormal DNA methylation, histone modifications, and miRNA expression can be observed in several types of CHD patients (Wang et al., 2022; Jarrell et al., 2019).

Long non-coding RNAs (lncRNAs) interact with chromatin through various mechanisms. These include direct interactions with transcription factors, chromatin-modifying complexes, and DNA. In this way, lncRNAs can participate in regulating the expression of key genes involved in heart development. In contrast, lncRNAs are abnormally expressed in patients with congenital heart disease, leading to heart structural defects (Rizki and Boyer, 2015). Interestingly, multiple studies have shown that the interactions between lncRNAs and chromatin can be detected and screened via Hi-C technology.

Hi-C can also detect more epigenetic modifications in CHD research. Biola M Javierre et al. used Hi-C technology to elucidate the influence of the primary cellular promoter interactome and to uncover regulatory mechanisms within genomes associated with prevalent diseases (Javierre et al., 2016). Chun Su et al. used single-cell RNA sequencing, single-cell ATAC sequencing, and high-resolution Hi-C on isolated cell populations to create detailed transcriptomic and 3D epigenomic portraits of human pancreatic acinar, alpha, and beta cells. This approach unveiled variations in A/B compartmentalization of chromatin, chromatin looping, and transcription factor-driven orchestration of cell-specific gene regulatory networks (Su et al., 2022).

### Hypertension

Blood pressure (BP) is considered a heritable characteristic, with approximately 30% of its variability linked to genetic components (Poulter et al., 2015). Primary hypertension predominantly affects individuals in middle to advanced age, stemming from the interplay of lifestyle choices and genetic predispositions (Poulter et al., 2015). Primary hypertension is characterized by its intricate genetic makeup and varied manifestations. While there is a wealth of genome-wide association research on hypertension, investigations into specific genetic loci and their interactions, particularly synergistic effects among multiple loci, remain limited (Loscalzo, 2019).

Research conducted on human subjects has indicated that fluctuations in BP and the onset of hypertension are correlated with alterations in DNA methylation patterns (Liang, 2018). Alterations in histone marks and the activity of long noncoding RNAs also contribute to the modulation of BP (Pentz et al., 2008; Pentz et al., 2012; Yu and Wang, 2018).

Variations in methylation patterns are known to impact the onset and progression of conditions such as hypertension and atherosclerosis (Toghill et al., 2015). Detecting disparities in DNA methylation patterns between individuals with hypertension and those with normal blood pressure indicates that shifts in DNA methylation could significantly contribute to the development of hypertension (Wang et al., 2013). With the advancement of Hi-C technology, derivative techniques are now capable of detecting DNA methylation. Studies have utilized this technology to accurately predict methylation levels within patients’ bodies. DNA methylation is also closely related to the development of hypertension, so Hi-C may detect methylation to monitor the occurrence of hypertension. Currently, this is still a novel direction.

Dysregulated methylation of the gene responsible for angiotensin II receptor type 1A (AT1AR) within the hypothalamus results in increased expression of hypothalamic AT1AR and heightened renal sympathetic activity in offspring from mice fed protein-restricted diets during pregnancy, which can lead to salt-sensitive hypertension (Kawarazaki and Fujita, 2021). In a rat model exposed to lipopolysaccharide (LPS) during gestation, prenatal exposure to LPS compromised the capacity to eliminate a sodium challenge and provoked hypertension in the first three generations. In the fourth and fifth generations, this exposure pattern subsequently leads to the development of hypertension, which is sensitive to dietary salt intake (Cao et al., 2022). Hypomethylation of the Klotho gene reduces the levels of circulating soluble Klotho, triggering vasoconstriction and other physiological responses that result in salt-sensitive hypertension due to a reduction in renal blood flow (Fujita, 2023). A recently developed graph transformer model called scHiMe can precisely forecast base-pair resolution (bp-resolution) methylation profiles via both single-cell Hi-C datasets and DNA sequence information (Zhu et al., 2023). The newly engineered scHiMe graph transformer model has demonstrated the ability to accurately predict methylation levels at the base-pair scale, utilizing single-cell Hi-C data and genomic DNA sequences (Zhu et al., 2023). Combining Hi-C technology with other technologies, such as scHiMe and ChIP-seq, can analyze the methylation of the genome more efficiently.

Vascular structures consist of three distinct layers: the intima, media, and adventitia. Endothelial cells line the intima, and smooth muscle cells are found in the media. These cells are the primary contributors to the functionality of blood vessels (Ma et al., 2023). Dysfunction of the endothelium is a common factor linked to high blood pressure and its associated complications (Silva et al., 2016). In rats with streptozotocin-induced diabetes and nitric oxide-deficient hypertension, intracellular changes in endothelial cells have been detected in the heart, indicating potential damage to capillary function along with adaptive mechanisms (Okruhlicova et al., 2005). Therefore, detecting the functional integrity of vascular endothelial cells is of great clinical importance for predicting the development of hypertension. Sirtuin 6, a widely conserved NAD-dependent deacetylase enzyme, protects against hypertension and its associated complications by preserving endothelial integrity through epigenetic mechanisms (Guo et al., 2019). By integrating Hi-C, DNase-Seq, ChIP-Seq, and RNA-Seq datasets, Jie Lv et al. and colleagues reported that MECOM interacts with enhancers to shape chromatin loops, thereby regulating genes essential for endothelial cell identity (Lv et al., 2023).

## Discussion

Hi-C technology not only resolves the 3D chromatin structure with high resolution but also helps us understand various substructures within it. A wealth of experimental data from modern medicine indicates that the occurrence of diseases is closely linked to changes in three-dimensional chromatin structure. Exploring the three-dimensional structure of chromatin is highly important for disease analysis, thereby advancing clinical medical diagnostic techniques and the research and development of drugs related to genomics. Many scientists have discovered new therapeutic targets for diseases via this technology (Table 1). Via Hi-C technology, drug developers can identify genes and regulatory elements related to cardiovascular diseases. Specifically, as mentioned earlier, Hi-C technology can detect interactions between genomic regions associated with diseases, helping to identify potential target genes. In addition, developers can combine Hi-C with other technologies (such as ChIP-seq) to verify the function of genomic regions identified by GWAS, thus determining the target genes that may be regulated. Furthermore, abnormalities in chromatin structure can serve as potential drug targets. Hi-C technology can provide clues to reveal such drug targets.

**TABLE 1:** Summary of the discovery of Hi-C-based therapeutic targets.

Study	Model	Hi-C application	Novel targets or site of detection	Epigenetic mechanisms
Rosa-Garrido et al. (2017)	Mice with heart-specific deletion of CTCF	To determine the endogenous structure of the cardiac myocyte epigenome	CTCF (a ubiquitous chromatin structural protein)	Pressure overload or CTCF depletion remodeled long-range interactions of cardiac enhancers
Arvanitis et al. (2020)	CRISPR-Cas9 enhancer deletion cardiomyocyte model	To identify and replicate associations between heart failure and one known locus in chromosome 4 near the PITX2 gene and two novel loci near the ABO (chromosome 9) and ACTN2 (chromosome 1) genes	Chromosome 4 near the PITX2 gene two novel loci near the ABO (chromosome 9) ACTN2 (chromosome 1) genes	A cardiac muscle-specific regulatory element that is dynamic during cardiomyocyte differentiation and binds to the promoter of the ACTN2 gene
Sabino et al. (2022)	Serum from anti-T positive patients	To detect that the newly associated locus interacts with chromatin sites in a number of different cell types and tissues	A new susceptibility locus for CCC.	*T cruzi* infects individuals through the modulation of a downstream transcriptional and protein signature associated with host-parasite immune response
Bertero et al. (2019)	hiPSC-CMs	To performed genome-wide chromosome conformation analyses in hiPSC-CMs with a haploinsufficient mutation for lamin A/C	A haploinsufficient LMNA mutation	A/B compartmentalization does not represent the primary mechanism directly leading to gene expression changes and disease pathogenesis
Zheng et al. (2018)	Hidden Markov model (HMM)	To detect the lamins interactions among the topologically associated chromatin domains (TADs)	Lamin-TKO mESCs	The lamin meshwork orchestrates 3D genome organization by differentially regulating different LADs, which in turn contributes to maintaining TAD-TAD interactions and interactions among chromatins with different activities
Javierre et al. (2016)	Human Primary Blood Cell Types	To detect interaction landscape of the INPP4B, RHAG, ZEB2-AS, and ALAD promoters in naive CD4 cells (nCD4)	22,076 fragments containing 31,253 annotated promoters	Promoter interactions are highly cell type specific and enriched for links between active promoters and epigenetically marked enhancers
Su et al. (2022)	Acinar, alpha, and beta cells	General validation of transcriptomic, chromatin accessibility and 3D architecture profiles in human pancreas cell subsets	Acinar: CPA1 and PRSS1; alpha: GCG and ARX; beta: INS and MAFA.	Differential A/B (open/closed) chromatin compartmentalization, chromatin looping, and transcription factor mediated control of cell type-specific gene regulatory programs
Guo et al. (2019)	Desoxycorticosterone acetate/salt-induced and Ang II (angiotensin II)-induced hypertensive mice	—	SIRT6 (sirtuin 6), one member of SIRTs (class III HDAC)	SIRT6 induced the expression of GATA5 (GATA-binding protein 5), a novel regulator of blood pressure, through inhibiting Nkx3.2 (NK3 homeobox 2) transcription by deacetylating histone H3K9 (histone H3 lysine 9), thereby regulating GATA5-mediated signaling pathways to prevent endothelial injury
Lv et al. (2023)	22 genome-wide chromatin marks in human umbilical vein endothelial cells (HUVEC)	To investigate the interaction between MECOM locus and its associated enhancers	MECOM binding motifs in the genome	The binding of MECOM on enhancers to regulate the transcription of target genes will trigger chromatin interactions between the enhancers and target genes

Hi-C technology provides more information than traditional high-throughput sequencing technology does. While high-throughput sequencing (NGS) can only assemble genomes at the contig/scaffold level, it fails to provide genome information at the chromosome level. In contrast, Hi-C assembly technology can place contigs/scaffolds onto different chromosomes, thereby enhancing genome quality.

Many emerging computational tools or machine learning approaches are enhancing the analysis of Hi-C data. For example, Qiao Liu et al. proposed scDEC-Hi-C. This is a new framework for single-cell Hi-C analysis with deep generative neural networks. It can comprehensively analyze sparse and heterogeneous single-cell Hi-C data (Diagnosis and classification of diabetes mellitus, 2013). In the report by Mattia Forcato et al., thirteen computational methods for Hi-C data analysis were detailed and compared, including algorithms for identifying chromatin interactions and algorithms for identifying topologically associating domains (TADs) (Forcato et al., 2017).

Since the prerequisite for using Hi-C technology is to obtain users’ genomic data, ensuring the privacy and security of users’ genetic data is essential. Moreover, researchers should ensure that the process of obtaining genomic information is compliant and that the data will not be leaked or misused. Accordingly, researchers should fulfill their duty to provide information, ensuring that users are informed and consenting throughout the process. Users should be aware of the purpose, methods, and potential risks of genomic research. When sharing research data, consent from users must be obtained first, ensuring that the entire process is conducted under strict legal and ethical frameworks. In gene therapy, the principle of treatment should be prioritized rather than economic benefits or scientific interests.

Hi-C technology is still in the development stage. Although it has a wide range of applications, it is not widely used at present. The main reasons are as follows: long experimental period, low correlation with high-resolution microscope results, high experimental threshold, difficulty of data analysis, low signal-to-noise ratio and low resolution. When Hi-C is performed with additional cross-linkers and/or with restriction enzymes that produce longer fragments, quantitative differences in cell type-specific chromosome organization can be missed or underestimated, depending on the 3C-based protocol used (Akgol et al., 2021). These factors also make it difficult for the Hi-C technique to detect weak chromatin interactions.

A kit can detect only one set of chromatin, and a single kit can cost as much as 50,000–80,000 RMB. To verify this conclusion, many repeated tests, which can be very expensive and involve hundreds of kits, are often needed. The experimental cost is very high, so few laboratories are involved in this field. The high threshold of Hi-C technology is also reflected in the high requirements for biological and statistical knowledge during operation. Understanding and skillfully operating Hi-C technology requires a certain understanding of the advantages and disadvantages of various statistical algorithms and knowing how to analyze the output results.

In many cases, Hi-C technology can be used to assess the rationality of new potential target genes, especially those far from disease-related SNPs. However, it is difficult to prove a gene’s role in a disease via only Hi-C technology. For example, consider laminopathies. For years, disruption of chromatin organization has been suspected to be a key part of disease pathogenesis. This is a very clear case. However, identifying and clearly identifying the pathogenic mechanism involving chromatin organization is still difficult. Moreover, other methods have been developed. They are used to identify related target genes and regulatory mechanisms that may involve 3D chromatin structure, such as epigenomic quantitative trait loci (eQTLs), mQTLs, etc.,, etc. and CRISPR-related technologies (CRISPR activation, inhibition, etc.). The use of Hi-C technology alone cannot provide strong evidence to prove the existence of regulatory mechanisms. It needs to be combined with CRISPR-related technologies and other methods.

To make Hi-C technology more widely used in cardiovascular research, the most important step is to reduce the cost of Hi-C technology. Researchers can lower the cost of using Hi-C by optimizing the technical workflow, updating relevant equipment, or using more economical reagents, making this technology more accessible to more laboratories. Moreover, simpler data algorithms and more efficient automated tools should be developed to reduce the complexity and time cost of experiments, improving their reproducibility and efficiency. The development of visualization software and artificial intelligence analysis tools can also help researchers process and analyze Hi-C data more efficiently.

Therefore, future research on this technology could focus on improving Hi-C technology, reducing production costs, increasing the utilization rate and reuse rate of reagents, reducing development and use costs, simplifying the experimental process, and improving the machine algorithm. If these issues are addressed, Hi-C technology could be expanded to the clinical market, not only limited to the laboratory but also becoming another powerful tool in the fight against disease.

## References

[B1] Akgol O. B. Yang L. Abraham S. Venev S. V. Krietenstein N. Parsi K. M. (2021). Systematic evaluation of chromosome conformation capture assays. Nat. Methods 18, 1046–1055. 10.1038/s41592-021-01248-7 34480151 PMC8446342

[B2] Arvanitis M. Tampakakis E. Zhang Y. Wang W. Auton A. Dutta D. (2020). Genome-wide association and multi-omic analyses reveal ACTN2 as a gene linked to heart failure. Nat. Commun. 11, 1122. 10.1038/s41467-020-14843-7 32111823 PMC7048760

[B3] Bertero A. Fields P. A. Smith A. S. T. Leonard A. Beussman K. Sniadecki N. J. (2019). Chromatin compartment dynamics in a haploinsufficient model of cardiac laminopathy. J. Cell Biol. 218, 2919–2944. 10.1083/jcb.201902117 31395619 PMC6719452

[B4] Burnett J. R. Hooper A. J. (2018). PCSK9 - a journey to cardiovascular outcomes. N. Engl. J. Med. 379, 2161–2162. 10.1056/NEJMe1813758 30485782

[B5] Cao N. Lan C. Chen C. Xu Z. Luo H. Zheng S. (2022). Prenatal lipopolysaccharides exposure induces transgenerational inheritance of hypertension. Circulation 146, 1082–1095. 10.1161/CIRCULATIONAHA.122.059891 36004643 PMC9529859

[B6] Carey R. M. Moran A. E. Whelton P. K. (2022). Treatment of hypertension: a review. JAMA 328, 1849–1861. 10.1001/jama.2022.19590 36346411

[B7] Dekker J. (2008). Gene regulation in the third dimension. Science 319, 1793–1794. 10.1126/science.1152850 18369139 PMC2666883

[B8] Dekker J. Rippe K. Dekker M. Kleckner N. (2002). Capturing chromosome conformation. Sci. Am. Assoc. Adv. Sci. 295, 1306–1311. 10.1126/science.1067799 11847345

[B9] Diagnosis and classification of diabetes mellitus (2013). Diagnosis and classification of diabetes mellitus. Diabetes Care 36 (Suppl. 1), S67–S74. 10.2337/dc13-S067 23264425 PMC3537273

[B10] Fatkin D. MacRae C. Sasaki T. Wolff M. R. Porcu M. Frenneaux M. (1999). Missense mutations in the rod domain of the lamin A/C gene as causes of dilated cardiomyopathy and conduction-system disease. N. Engl. J. Med. 341, 1715–1724. 10.1056/NEJM199912023412302 10580070

[B11] Feng Y. Huang W. Paul C. Liu X. Sadayappan S. Wang Y. (2021a). Mitochondrial nucleoid in cardiac homeostasis: bidirectional signaling of mitochondria and nucleus in cardiac diseases. Basic Res. Cardiol. 116, 49. 10.1007/s00395-021-00889-1 34392401 PMC8364536

[B12] Feng Y. Liu X. Pauklin S. (2021b). 3D chromatin architecture and epigenetic regulation in cancer stem cells. Protein Cell 12, 440–454. 10.1007/s13238-020-00819-2 33453053 PMC8160035

[B13] Feng Y. Pauklin S. (2020). Revisiting 3D chromatin architecture in cancer development and progression. Nucleic Acids Res. 48, 10632–10647. 10.1093/nar/gkaa747 32941624 PMC7641747

[B14] Forcato M. Nicoletti C. Pal K. Livi C. M. Ferrari F. Bicciato S. (2017). Comparison of computational methods for Hi-C data analysis. Nat. Methods 14, 679–685. 10.1038/nmeth.4325 28604721 PMC5493985

[B15] Fujita T. (2023). Recent advances in hypertension: epigenetic mechanism involved in development of salt-sensitive hypertension. Hypertension 80, 711–718. 10.1161/HYPERTENSIONAHA.122.20588 36583382

[B16] Gao X. Zheng P. Yang L. Luo H. Zhang C. Qiu Y. (2019). Association of functional variant in GDF1 promoter with risk of congenital heart disease and its regulation by Nkx2.5. Clin. Sci. (Lond). 133, 1281–1295. 10.1042/CS20181024 31171573

[B17] Godfraind T. (2014). Calcium channel blockers in cardiovascular pharmacotherapy. J. Cardiovasc Pharmacol. Ther. 19, 501–515. 10.1177/1074248414530508 24872348

[B18] Goodwin S. McPherson J. D. McCombie W. R. (2016). Coming of age: ten years of next-generation sequencing technologies. Nat. Rev. Genet. 17, 333–351. 10.1038/nrg.2016.49 27184599 PMC10373632

[B19] Guo J. Wang Z. Wu J. Liu M. Li M. Sun Y. (2019). Endothelial SIRT6 is vital to prevent hypertension and associated cardiorenal injury through targeting nkx3.2-GATA5 signaling. Circ. Res. 124, 1448–1461. 10.1161/CIRCRESAHA.118.314032 30894089

[B20] Jarrell D. K. Lennon M. L. Jacot J. G. (2019). Epigenetics and mechanobiology in heart development and congenital heart disease. Diseases 7, 52. 10.3390/diseases7030052 31480510 PMC6787645

[B21] Javierre B. M. Burren O. S. Wilder S. P. Kreuzhuber R. Hill S. M. Sewitz S. (2016). Lineage-specific genome architecture links enhancers and non-coding disease variants to target gene promoters. Cell 167, 1369–1384.e19. 10.1016/j.cell.2016.09.037 27863249 PMC5123897

[B22] Kawarazaki W. Fujita T. (2021). Kidney and epigenetic mechanisms of salt-sensitive hypertension. Nat. Rev. Nephrol. 17, 350–363. 10.1038/s41581-021-00399-2 33627838

[B23] Liang M. (2018). Epigenetic mechanisms and hypertension. Hypertension 72, 1244–1254. 10.1161/HYPERTENSIONAHA.118.11171 30571238 PMC6314488

[B24] Ling C. Rönn T. (2019). Epigenetics in human obesity and type 2 diabetes. Cell Metab. 29, 1028–1044. 10.1016/j.cmet.2019.03.009 30982733 PMC6509280

[B25] Loscalzo J. (2019). Precision medicine. Circ. Res. 124, 987–989. 10.1161/CIRCRESAHA.119.314403 30920923 PMC6501802

[B26] Lv J. Meng S. Gu Q. Zheng R. Gao X. Kim J.-D. (2023). Epigenetic landscape reveals MECOM as an endothelial lineage regulator. Nat. Commun. 14, 2390. 10.1038/s41467-023-38002-w 37185814 PMC10130150

[B27] Ma J. Li Y. Yang X. Liu K. Zhang X. Zuo X. (2023). Signaling pathways in vascular function and hypertension: molecular mechanisms and therapeutic interventions. Signal Transduct. Target Ther. 8, 168. 10.1038/s41392-023-01430-7 37080965 PMC10119183

[B28] Martin P. Ding J. Duffus K. Gaddi V. P. McGovern A. Ray-Jones H. (2019). Chromatin interactions reveal novel gene targets for drug repositioning in rheumatic diseases. Ann. Rheum. Dis. 78, 1127–1134. 10.1136/annrheumdis-2018-214649 31092410 PMC6691931

[B29] McGettigan P. Ferner R. E. (2017). PCSK9 inhibitors for hypercholesterolaemia. BMJ 356, j188. 10.1136/bmj.j188 28104607

[B30] Mewborn S. K. Puckelwartz M. J. Abuisneineh F. Fahrenbach J. P. Zhang Y. MacLeod H. (2010). Altered chromosomal positioning, compaction, and gene expression with a lamin A/C gene mutation. PLoS One 5, e14342. 10.1371/journal.pone.0014342 21179469 PMC3001866

[B31] Montefiori L. E. Sobreira D. R. Sakabe N. J. Aneas I. Joslin A. C. Hansen G. T. (2018). A promoter interaction map for cardiovascular disease genetics. Elife 7, e35788. 10.7554/eLife.35788 29988018 PMC6053306

[B32] Okruhlicova L. Tribulova N. Weismann P. Sotnikova R. (2005). Ultrastructure and histochemistry of rat myocardial capillary endothelial cells in response to diabetes and hypertension. Cell Res. 15, 532–538. 10.1038/sj.cr.7290322 16045816

[B33] Pentz E. S. Cordaillat M. Carretero O. A. Tucker A. E. Sequeira Lopez M. L. S. Gomez R. A. (2012). Histone acetyl transferases CBP and p300 are necessary for maintenance of renin cell identity and transformation of smooth muscle cells to the renin phenotype. Am. J. Physiol. Heart Circ. Physiol. 302, H2545–H2552. 10.1152/ajpheart.00782.2011 22523253 PMC3378259

[B34] Pentz E. S. Lopez MLSS Cordaillat M. Gomez R. A. (2008). Identity of the renin cell is mediated by cAMP and chromatin remodeling: an *in vitro* model for studying cell recruitment and plasticity. Am. J. Physiol. Heart Circ. Physiol. 294, H699–H707. 10.1152/ajpheart.01152.2007 18055510

[B35] Poulter N. R. Prabhakaran D. Caulfield M. (2015). Hypertension. Lancet. 386, 801–812. 10.1016/S0140-6736(14)61468-9 25832858

[B36] Risca V. I. Greenleaf W. J. (2015). Unraveling the 3D genome: genomics tools for multiscale exploration. Trends Genet. 31, 357–372. 10.1016/j.tig.2015.03.010 25887733 PMC4490074

[B37] Rizki G. Boyer L. A. (2015). Lncing epigenetic control of transcription to cardiovascular development and disease. Circ. Res. 117, 192–206. 10.1161/CIRCRESAHA.117.304156 26139858

[B38] Rosa-Garrido M. Chapski D. J. Schmitt A. D. Kimball T. H. Karbassi E. Monte E. (2017). High-resolution mapping of chromatin conformation in cardiac myocytes reveals structural remodeling of the epigenome in heart failure. Circulation 136, 1613–1625. 10.1161/CIRCULATIONAHA.117.029430 28802249 PMC5648689

[B39] Sabino E. C. Franco L. A. M. Venturini G. Velho Rodrigues M. Marques E. Oliveira-da Silva L. (2022). Genome-wide association study for Chagas Cardiomyopathy identify a new risk locus on chromosome 18 associated with an immune-related protein and transcriptional signature. PLoS Negl. Trop. Dis. 16, e0010725. 10.1371/journal.pntd.0010725 36215317 PMC9550069

[B40] Sheng-Shou Hu (2022). Report on cardiovascular health and diseases in China 2021: an updated summary. Biomed. Environ. Sci. 35, 573–603. 10.26599/1671-5411.2023.06.001 35945174

[B41] Silva G. C. Silva J. F. Diniz T. F. Lemos V. S. Cortes S. F. (2016). Endothelial dysfunction in DOCA-salt-hypertensive mice: role of neuronal nitric oxide synthase-derived hydrogen peroxide. Clin. Sci. (Lond). 130, 895–906. 10.1042/CS20160062 26976926

[B42] Su C. Gao L. May C. L. Pippin J. A. Boehm K. Lee M. (2022). 3D chromatin maps of the human pancreas reveal lineage-specific regulatory architecture of T2D risk. Cell Metab. 34, 1394–1409.e4. 10.1016/j.cmet.2022.08.014 36070683 PMC9664375

[B43] TifHMa E. (2021). Cardiovascular diseases — level 2 cause. Available at: https://www.healthdata.org/research-analysis/diseases-injuries-risks/factsheets/2021-cardiovascular-diseases-level-2-disease

[B44] Timmis A. Vardas P. Townsend N. Torbica A. Katus H. De Smedt D. (2022). European Society of Cardiology: cardiovascular disease statistics 2021. Eur. Heart J. 43, 716–799. 10.1093/eurheartj/ehab892 35016208

[B45] Toghill B. J. Saratzis A. Harrison S. C. Verissimo A. R. Mallon E. B. Bown M. J. (2015). The potential role of DNA methylation in the pathogenesis of abdominal aortic aneurysm. Atherosclerosis 241, 121–129. 10.1016/j.atherosclerosis.2015.05.001 25974102

[B46] van Berkum N. L. Lieberman-Aiden E. Williams L. Imakaev M. Gnirke A. Mirny L. A. (2010). Hi-C: a method to study the three-dimensional architecture of genomes. J. Vis. Exp., 1869. 10.3791/1869 20461051 PMC3149993

[B47] Varela D. Conceição N. Cancela M. L. (2021). Transcriptional regulation of human T-box 5 gene (TBX5) by bone- and cardiac-related transcription factors. Gene 768, 145322. 10.1016/j.gene.2020.145322 33221539

[B48] Wang G. Wang B. Yang P. (2022). Epigenetics in congenital heart disease. J. Am. Heart Assoc. 11, e025163. 10.1161/JAHA.121.025163 35348004 PMC9075469

[B49] Wang J. C. Bennett M. (2012). Aging and atherosclerosis: mechanisms, functional consequences, and potential therapeutics for cellular senescence. Circ. Res. 111, 245–259. 10.1161/CIRCRESAHA.111.261388 22773427

[B50] Wang X. Falkner B. Zhu H. Shi H. Su S. Xu X. (2013). A genome-wide methylation study on essential hypertension in young African American males. PLoS One 8, e53938. 10.1371/journal.pone.0053938 23325143 PMC3542324

[B51] Williams K. Carson J. Lo C. (2019). Genetics of congenital heart disease. Biomolecules 9, 879. 10.3390/biom9120879 31888141 PMC6995556

[B52] Yu B. Wang S. (2018). Angio-LncRs: LncRNAs that regulate angiogenesis and vascular disease. Theranostics 8, 3654–3675. 10.7150/thno.26024 30026873 PMC6037039

[B53] Yuan S. Zaidi S. Brueckner M. (2013). Congenital heart disease: emerging themes linking genetics and development. Curr. Opin. Genet. Dev. 23, 352–359. 10.1016/j.gde.2013.05.004 23790954 PMC4154700

[B54] Zheng M. Tian S. Z. Capurso D. Kim M. Maurya R. Lee B. (2019). Multiplex chromatin interactions with single-molecule precision. Nature 566, 558–562. 10.1038/s41586-019-0949-1 30778195 PMC7001875

[B55] Zheng X. Hu J. Yue S. Kristiani L. Kim M. Sauria M. (2018). Lamins organize the global three-dimensional genome from the nuclear periphery. Mol. Cell. 71, 802–815. 10.1016/j.molcel.2018.05.017 30201095 PMC6886264

[B56] Zhu H. Liu T. Wang Z. (2023). scHiMe: predicting single-cell DNA methylation levels based on single-cell Hi-C data. Brief. Bioinform 24, bbad223. 10.1093/bib/bbad223 37302805 PMC10359091

